# Mitochondrial DNA Pathogenic Variant Prevalence in Primary Mitochondrial Disease Patients With African (L) Mitochondrial Genome Haplogroups

**DOI:** 10.1002/jmd2.70036

**Published:** 2025-07-11

**Authors:** Surita Meldau, Elizabeth M. McCormick, Ibrahim George‐Sankoh, Gillian T. Riordan, Kashief Khan, Laura E. MacMullen, Shrinav Dawlat, Dee Blackhurst, Marni J. Falk, Joanna L. Elson

**Affiliations:** ^1^ National Health Laboratory Service Cape Town South Africa; ^2^ Division of Chemical Pathology, Department of Pathology University of Cape Town Cape Town South Africa; ^3^ Mitochondrial Medicine Frontier Program, Division of Human Genetics, Department of Pediatrics Children's Hospital of Philadelphia Philadelphia Pennsylvania USA; ^4^ Department of Bioinformatics Children's Hospital of Philadelphia Philadelphia Pennsylvania USA; ^5^ Division of Paediatric Neurology, Department of Paediatrics and Child Health Red Cross War Memorial Children's Hospital, University of Cape Town Cape Town South Africa; ^6^ Department of Pediatrics University of Pennsylvania Perelman School of Medicine Philadelphia Pennsylvania USA; ^7^ Biosciences Institute, Newcastle University Newcastle Upon Tyne UK; ^8^ Centre for Human Metabolomics North‐West University Potchefstroom South Africa

**Keywords:** African American, haplogroup context, haplogroup L, primary mitochondrial disease, South Africa

## Abstract

Primary mitochondrial diseases (PMD) are caused by pathogenic variants in over 350 genes, 37 of which are located in mitochondrial DNA (mtDNA). While more than 100 mtDNA variants have confirmed disease associations, there are few reports of mtDNA‐related PMD in patients with African heritage, even in well‐studied populations. We investigated the frequency of pathogenic mtDNA variants in African L‐haplogroups in patients with confirmed PMD from two diagnostic cohorts. Data from genetically confirmed mtDNA‐related cases were extracted from existing databases at the National Health Laboratory Service Inherited Metabolic Disease Laboratory in South Africa (SA), and the Children's Hospital of Philadelphia (CHOP) Mitochondrial Medicine Frontier Program (USA). Mitochondrial genome haplogroup context was recorded from existing sequence report data. Stored DNA from the remaining cases was sequenced for mitochondrial genome haplogroup determination. Haplogroup context was obtained for 82 SA and 165 CHOP PMD cases. Sixty‐two (47 SA; 15 USA) PMD cases from at least 50 maternal lineages were found to carry L Haplogroups. Unique L sub‐haplogroups were identified in 11 (9 SA, 2 USA) families with the m.3243A>G MELAS variant, 6 SA families with the m.11778G>A LHON variant, and 20 (15 SA, 5 USA) cases with single large‐scale mtDNA deletions (4 of whom had the 4977 bp common deletion). Several additional well‐documented mtDNA pathogenic variants were identified in L‐haplogroup context. PMD patient clinical features correlated closely with those described in other haplogroup cohorts. This study demonstrates that common pathogenic mtDNA variants occur in the context of multiple African mtDNA lineages. Disproportionately low diagnostic rates highlight ongoing diagnostic inequalities affecting those on the African continent and African patients globally.

**Summary**

- Common pathogenic mtDNA variants causal of PMD occur in the context of African mtDNA genome haplogroup lineages, while persistent diagnostic inequalities contribute to underdiagnosis of primary mitochondrial disease.

## 1 Introduction

Primary mitochondrial diseases (PMD) are a large group of genetically and phenotypically heterogeneous disorders arising from inherited defects in mitochondrial energy production. They account for a significant proportion of inherited metabolic defects worldwide [1], with an estimated prevalence of at least 1 in 4300 [2]. PMD can be caused by pathogenic variants in any one of more than 400 genes encoding mitochondrial proteins with proven gene‐disease associations, including genes involved in transcriptional, translational, and other regulatory pathways [3]. Thirty‐seven of these genes are located in the mitochondrial DNA (mtDNA) genome [1].

Very little is known about PMD on the African continent. Indeed, limited information is known about PMD in those with African ancestry globally. A few publications from South Africa in the extreme south of Africa [4, 5, 6, 7, 8, 9, 10, 11, 12, 13, 14, 15, 16, 17, 18], as well as in Tunisia [19, 20, 21, 22, 23, 24, 25, 26, 27, 28, 29, 30, 31, 32, 33, 34, 35, 36, 37, 38], Morocco [39, 40, 41, 42] and Egypt [43, 44, 45, 46] in the extreme north of Africa, have described isolated cohorts of patients with suspected PMD. However, descriptions of genetic defects underlying the disease are lacking for many of these cohorts. Genetically defined cases have mainly been in the context of nuclear DNA (nDNA) disease, with many reported resulting from consanguinity [19, 22, 23] and others due to founder variants [4, 11, 40] in isolated populations.

No cases of mitochondrial encephalomyopathy with lactic acidosis and stroke‐like episodes (MELAS) or maternally‐inherited diabetes and deafness (MIDD), or any overlap syndromes related to the common m.3243A>G variant in *MT‐TL1* have been reported in sub‐Saharan Africa, with the exception of an ethnically diverse South African cohort [6].

There is also a paucity of reports of confirmed mtDNA related PMD in patients with sub‐Saharan African heritage in the literature, even in well‐studied population cohorts such as those from North America [47, 48]. Possible explanations for this observation have included unknown masking factors and disease modifiers that may reduce penetrance conferred by haplogroup context [9, 47, 49, 50, 51]. Strong empirical evidence supporting these hypotheses remains lacking.

The mitochondrial DNA haplogroups associated with the African continent are denoted by the letter L and are found in individuals of Black African heritage globally. It is important to note that the haplogroups found in other global populations fall within the macrohaplogroups MN, which are a subgroup of the African haplogroup L3. Individuals who are categorized within the supergroup MN are not included in this study.

Only a handful of studies have ever reported on mtDNA disease occurring in the context of genetically confirmed African L haplogroups [6, 10, 12, 52, 53] which, in the absence of additional data, may support a hypothesis that mtDNA related disease less frequently occurs in African populations [9, 10], whether due to the influence of haplogroup context [54, 55] or other yet undefined mechanisms.

Meldau et al. recently reported on 155 genetically confirmed PMD cases from South Africa in a retrospective cohort study from a single diagnostic referral laboratory [6]. Although the largest of its kind from the African continent, this study evaluated largely unscreened referrals from the genetically and ethnically diverse South African population that includes many from European‐derived ancestries; little to no data was available on the ancestral diversity in the positive cases. Haplogroup context was reported for only eight of the 113 patients with mtDNA‐based PMD, of whom four had L haplogroups. Three of these individuals carried very rare mtDNA variants (m.3635G>A, m.10197G>A, m.13094T>C), while a single individual carried the well‐known Leigh syndrome spectrum variant, m.9176T>C, in the context of an L2 haplogroup [6].

This study aimed to identify and characterize a comprehensive cohort of PMD patients of African maternal ancestry with confirmed African L haplogroups in the context of pathogenic mtDNA variants. mtDNA genome sequencing was performed in South African patients with genetically confirmed PMD at a national referral laboratory that serves both state and private healthcare services, and in a large PMD cohort of individuals with mtDNA‐based diseases and African ancestry from the United States. This paper shows conclusively for the first time that the common disease‐associated variants often seen in European PMD patients are also seen in black African heritage. We also consider the possibility of diagnostic disparities and the possible reasons for this.

## 2 Methods

Institutional ethical approval for this study was obtained from the University of Cape Town (UCT) Human Research Ethics Committee (HREC #156/2021). Subjects from the United States were enrolled via an informed consent process or decedent waiver into Children's Hospital of Philadelphia (CHOP) Institutional Review Board (IRB) study #08‐6177 (Falk, PI; active 2008 to present), reflecting a national and international origin referral base.

The SA cohort represented most of the 96 families described in the original cohort publication from 2022 [6], and additional patients subsequently diagnosed. Data from all genetically‐confirmed mtDNA related PMD cases from SA were extracted from the National Health Laboratory Service (NHLS)/UCT Inherited Metabolic disease database for analysis. Where mtDNA genome sequencing was originally done for the diagnostic workup, haplogroup data was recorded (*N* = 24). Urine, muscle, or peripheral blood derived DNA specimens were obtained for the remainder of the positive cases (*N* = 74) from the UCT HREC registered Inherited Metabolic Disease repository. Sixteen samples were excluded from further analysis due to insufficient material and/or degraded DNA, as evidenced by repeated lack of PCR amplification. The complete coding region of the mtDNA was amplified in the remaining 58 cases, using the Qiagen UltraRun long‐range PCR kit (Qiagen, Germany), followed by library preparation using the Nexterra XT DNA library preparation kit (Illumina, USA), and sequencing using the Illumina MiSeq v.2 Nano or Micro kits (Illumina, USA). MtDNA variants were called using mtDNA‐Server [56], which includes haplogroup analysis through Haplogrep [57]. Where family relationships were clearly established, sequencing was only performed on 1 maternal family member, with the haplogroup of the other positive cases in the same family inferred.

mtDNA genome sequencing was performed on a clinical diagnostic basis in individuals with suspected PMD in the US cohort. Genetic testing reports were reviewed and haplogroup information extracted. In several instances where haplogroups were not provided but benign variants were listed on clinical testing reports, mtDNA genome sequence variants were entered into MITOMASTER SNV query tool to obtain haplogroup information [58]. Available clinical phenotype data, as well as heteroplasmy levels of reported variants were collated and major phenotypes described.

Statistical analysis on the proportion differences between the cohorts and population figures was performed using a one‐sample Z‐test for proportions conducted in GraphPad Prism v10.

## 3 Results

Haplogroup information was obtained from existing and newly generated next‐generation sequencing (NGS) data for 82 SA cases, of which 67 carried unique mutation‐haplogroup combinations that suggested a minimum of 67 families were represented in the entire cohort. Forty‐seven of these 82 cases (57%) had African L haplogroups. NGS data analysis confirmed the presence of all pathogenic variants previously identified through alternative methodologies (RFLP and/or Sanger sequencing for single variants). In addition, 15 out of 165 (9%) US PMD patients, for whom haplogroup data was accessible, representing a total of 12 unique kindreds, were found to have L haplogroup ancestry and pathogenic mtDNA variants.

Combined data revealed unique African (L) haplogroups for at least 11 (9 SA, 2 USA) families with the common m.3243A>G MELAS/MIDD variant (Table 1), 6 (6 SA, 0 USA) families with the common m.11778G>A LHON variant (Table 2), 20 (15 SA, 5 USA) patients with single large‐scale mtDNA deletions (SLSMD, of which 4 SA patients carried the common 4977 bp mtDNA deletion, Table 3), 2 (0 SA, 2 USA) families with the common m.8993T>G NARP variant, 2 (0 SA, 2 USA) families with the common m.13513G>A Leigh syndrome spectrum variant, and one PMD family each with m.14484T>C, m.3460G>A, m.9176T>C, m.8993T>C, m.3635G>A, m.8344A>G, m.10197G>A, m.13094T>C, and m.14453G>A pathogenic mtDNA variants (Table 4). Complete details are summarized for each PMD patient of their mtDNA genome haplogroup data, mtDNA pathogenic variant heteroplasmy level, demographics, and available clinical phenotype data for all L haplogroup cases (Tables 1, 2, 3, 4).

**TABLE 1:** Clinical presentation and demographics of patients shown to carry the *NC_012920.1(MT‐TL1*):m.3243A>G variant in the context of African L‐haplogroups identified in this study.

Patient ID	Cohort	Age at genetic diagnosis	Sex	Mito haplogroup	Heteroplasmy level	Eye signs	SLE	Seizures	Dementia/Neuro regression	Cerebellar signs	SNHL	DM	Myopathy	CMO	Heart Block	Other key clinical features	Syndrome reported
10	SA	25 years	M	L2a1b1a^a^	86.9% urine		+	+								DD, lactatemia	
12	SA	32 years	F		57.7% urine						+	+					MIDD
13	SA	7 years	M		71.9% blood		+		+							Elevated CSF lactate; DD; FTT	
16	SA	13 years	M		62.3% blood			+							+		
11	SA	24 years	F	L0d1a1a1	46.2% blood		+									Abnormal balance, behavioral changes	
9	SA	29 years	F	L2a1f	51.8% urine	Bilateral vision loss	+	+			+					Family history: sister had stroke at 26 years	MELAS
14	SA	42 years	M	L0a2a2a	89.7% Muscle		+	+									
15	SA	32 years	M	L0d2a1a	44.9% blood		+	+	+								
17	SA	15 years	M	L2a1a	62.4% blood											NCH	
18	SA	18 years	M	L3e1b2	63.9% blood		+	+			+						
19	SA	16 years	M	L0d2a1a1	91.5% Muscle	Optic atrophy	+									Short stature, mother is deaf	
20	SA	45 years	F	L3e1a3a	84.8% Urine					+	+	+				Hypercholesterolaemia, hypertension	MIDD
48	USA	7 years	M	L2a1f3^b^	77% blood		+	+	+	+	+		+	+		Short stature, FTT, WPW	MELAS
49	USA	46 years	F		< 10% blood											Migraines	
53	USA	28 years	F	L3d3a	5% blood, 5% buccal, 6% urine											Fasting intolerance, headaches, mood disorder, family history of PMD	

Abbreviations: CMO, cardiomyopathy; CSF, cerebrospinal fluid; DD, developmental delay; DM, diabetes mellitus; FTT, failure to thrive; MELAS, mitochondrial encephalomyopathy with lactic acidosis and stroke‐like episodes; MIDD, maternally‐inherited diabetes and deafness; NCH, no clinical history; PMD, primary mitochondrial disease; SA, South African Cohort; SLE, stroke‐like episodes; SNHL, sensorineural hearing loss; USA, United States Cohort; WPW, Wolff–Parkinson–White arrhythmia.

^a^ Presumed familial relationship based on haplogroups identified with the same genetic defect.

^b^ Known familial relationship.

**TABLE 2:** Clinical presentation and demographics of patients shown to carry the *NC_012920.1(MT‐ND4):m.11778G>A* variant in the context of African L‐haplogroups identified in this study.

Patient ID	Cohort	Mito haplogroup	Heteroplasmy level	Age at genetic diagnosis	Age at onset (if known)	Sex	Eye signs	Syndrome reported
1	SA	L0d2a1a2^a^	99.8% blood	15 years	12 years	M	Optic atrophy	LHON
3	SA		99.7% blood	55 years	u/k	M	Optic atrophy	LHON
5	SA	L0d2a1a^a^	99.8% blood	16 years	16 years	M	Optic atrophy	LHON
8	SA		93.1% blood	34 years	u/k	M	Optic atrophy	
2	SA	L0a2a2a	99.9% blood	17 years	u/k	M	Optic neuritis	
4	SA	L0d1b2b2b	99.9% blood	34 years	u/k	M		LHON
6	SA	L3d1a1a1	100% blood	35 years	u/k	M		LHON
7	SA	L3e2b1a2	99.8% blood	29 years	27 years	M	Optic atrophy	LHON

Abbreviation: LHON, Leber's hereditary optic neuropathy.

^a^ Presumed familial relationship based on haplogroups identified with the same genetic defect.

**TABLE 3:** Clinical presentation and demographics of patients shown to carry single large‐scale mtDNA deletions (SLSMD) in the context of African L‐haplogroups identified in this study.

Patient ID	Cohort	Age at genetic diagnosis	Sex	Result	Mito haplogroup	Eye signs	Cerebellar signs	SNHL	DM	Myopathy	CMO	Heart Block	Other Key clinical features	Syndrome reported	No clinical history available
26	SA	23 years	M	m.8482_13460del (CD)	L0a1b2a	CPEO RP Ptosis				+					
27	SA	41 years	F	m.8482_13460del (CD)	L2a1b1a	CPEO									
28	SA	25 years	M	m.8482_13460del (CD)	L0d2a1									KSS	
29	SA	23 years	M	m.8482_13460del (CD)	L0d1b2b2c	CPEO									
30	SA	10 years	F	m.7128_14006del	L1c2a3a	CPEO RP Ptosis	+								
31	SA	34 years	F	m.7737_15605del	L0d2b1a	CPEO RP	+			+					
32	SA	36 years	F	m.10946_15363del	L2a1a									KSS	
33	SA	9 months 18 days	M	m.10050_15438del	L3e3b1									Pearson	
34	SA	22 years	M	m.11003_15527del	L0d2c2	CPEO RP Ptosis				+			Short stature	KSS	
35	SA	52 years	M	m.11607_15438del	L3e2b1a2	CPEO Ptosis			+				Dyslipidemia, hypertension		
36	SA	15 years	M	m.7824_15388del	L0d2c2									KSS	
37	SA	22 years	F	m.8565_15607del	Lod2a1	Ptosis RP	+	+				+	RRF in muscle		
38	SA	49 years	F	m.9497_13835del	L0d1b2b1b1	CPEO		+			+		Dysphagia	KSS	
39	SA	70 years	F	m.12113_144221del	L0d2c2	CPEO				+			Cox negative fibers and RRF in muscle		
40	SA	6 years	M	m.8623_15656del	L0a1b1a1	RP							FTT	Fanconi S	
56	USA	18 years	F	m.5787_16075del (and m.10038G>A)	L3f1b3	Retinal dystrophy, cataracts	+	+		+			FTT, chronic kidney disease, Cox negative fibers and RRF in muscle	MM	
57	USA	4 years	M	m.695_2449del	L2c2b1a	Nyctalopia, normal fundus exam, mild changes noted on ERG	+			+			Migraines; FTT; ADHD, Immune deficiency	Pearson	
59	USA	12 years	M	m.3566_15537del	L2a1e1	Optic atrophy, ophthalmoplegia	+	+		+	+ (LVH)	No (ICD placed)	Migraines, hypertension, mitral valve prolapse	KSS	
60	USA	8 months	M	m.10668_14230del	L3d1d								SGA/IUGR at birth, short stature, FTT	Pearson	
62	USA	22 months	M	m.6456_11401del	L2c1								^a^		

Abbreviations: ADHD, attention deficit hyperactivity disorder; CD, common 4977bp deletion; CMO, cardiomyopathy; CPEO, chronic progressive external ophthalmoplegia; DD, developmental delay; DM, diabetes mellitus; ERG, electroretinogram; Fanconi S, Fanconi syndrome; FTT, failure to thrive; ICD, implantable cardioverter defibrillator; IUGR, intrauterine growth restriction; KSS, Kearns Sayre syndrome; LVH, left ventricular hypertrophy; MELAS, mitochondrial encephalomyopathy with lactic acidosis and stroke‐like episodes; MIDD, maternally‐inherited diabetes and deafness; MM, mitochondrial myopathy; RP, retinitis pigmentosa; RRF, ragged red fibers; SGA, small for gestational age; SLE, stroke‐like episodes; SNHL, sensorineural hearing loss.

^a^ Born premature at 26 weeks, however it is currently unclear if manifestations of developmental delay, seizures, and/or feeding difficulty are related to prematurity or mitochondrial DNA deletion.

**TABLE 4:** Clinical presentation and demographics of patients shown to carry other less common, yet well‐defined pathogenic mtDNA variants in the context of African L‐haplogroups identified in this study.

Patient ID	Cohort	Age at genetic diagnosis	Sex	Pathogenic variant	Heteroplasmy level	Mito haplogroup	Eye signs	SLE	Seizures	Dementia/Neuroregression	Cerebellar signs	SNHL	Myopathy	CMO	Other key clinical features	Syndrome reported
41^a^	SA	3 years	M	MT‐ATP6:m.9176T>C	100% muscle	L2a1a	Nystagmus								Respiratory problem, Raised CSF lactate	LSS
42	SA	52 years	F	MT‐ATP6:m.8993T>C	64.3% blood	L0d1b2b2b1									Strong family history	NARP
43	SA	26 years	M	MT‐ND1:m.3460G>A	14.3% blood	L3e1b2	Chronic papilledema									LHON^c^
44^d^	SA	13 years	F	MT‐ND1:m.G3635A	100% muscle	L0a1b1a1	CPEO Ptosis					+	+	+		
45^d^	SA	7 years	M	MT‐ND3:m.10197	100% blood	L1c2a3a										LSS
46^d^	SA	2 years 8 months	M	MT‐ND5:m.13094T>C	83% muscle	L0d1c1a	Optic atrophy ophthalmoplegia				+					LSS
47	SA	39 years	F	MT‐ND6:m.14453G>A	87% muscle, < 5% blood	L0d1a1a1		+	+							MELAS
21	SA	27 years	M	MT‐ND6:m.14484T>C^a^	100% blood	L2a1b1a										LHON
22	SA	21 years	M		100% blood											LHON
23	SA	16 years	?M		100% blood		Central visual field loss									LHON
24	SA	29 years	M		100% blood		Optic atrophy									
25	SA	u/k	M		98.9% blood										NCH	
50	USA	16 months	F	MT‐TK:m.8344A>G^b^	97% blood	L2c2	Optic atrophy		+	+	+		+		Respiratory failure	LSS/MERRF overlap
51	USA	44 years	F		88% blood				+	+	+	+	+		Lipomas	MERRF
52	USA	39 years	F		79% blood				+						Lipoma	
54	USA	6 years	F	MT‐ND5:m.13513G>A	45% muscle	L2a1a3c	Optic atrophy		+	+	+	+	+		FTT	LSS
61	USA	17 months	M		60% buccal	L3f1b1a	Ptosis					+			Short stature/FTT, dystonia, hyperreflexia	
55	USA	7 months	M	MT‐ATP6:m.8993T>G	98% blood	L2a1c4a1	Optic atrophy		+	+	+				FTT, lactic acidosis	LSS
58	USA	4 months	M		96% blood	L2a1	Optic atrophy		+				+	+ (mild LVH)	Feeding difficulty, choreiform movements	LSS

Abbreviations: CMO, cardiomyopathy; CPEO, chronic progressive external ophthalmoplegia; DM, diabetes mellitus; FTT, failure to thrive; LHON, Leber's hereditary optic neuropathy; LSS, Leigh syndrome spectrum; LVH, left ventricular hypertropy; MELAS, mitochondrial encephalomyopathy with lactic acidosis and stroke‐like episodes; MERRF, myoclonic epilepsy with ragged red fibers; MIDD, maternally inherited diabetes and deafness; NARP, neurogenic muscle weakness, ataxia, and retinitis pigmentosa; NCE, no clinical history; SLE, stroke‐like episodes; SNHL, sensorineural hearing loss.

^a^ Presumed familial relationship based on haplogroups identified with the same genetic defect.

^b^ Known familial relationship.

^c^ Possible incidental finding (very low heteroplasmy).

^d^ Previously described.

## 4 Discussion

Here, we described the first comprehensive retrospective cohort study of mtDNA haplogroup and variant characteristics in African ancestry patients across a major diagnostic referral laboratory in South Africa and a major clinical center in the United States. This work clearly demonstrates that known pathogenic variants in the mtDNA genome that are causal of classical mitochondrial disease pathology do occur at a much greater degree than previously suggested across 97 PMD patients from multiple African mtDNA genome lineages. Collectively, the common m.3243A>G MELAS/MIDD variant, and m.11778G>A LHON variant, as well as single large‐scale mtDNA deletions (SLSMDs) accounted for most (47%, 37 of 79 unique kindreds) of the PMD cases described, while various other well‐recognized pathogenic variants were identified in the African L macrohaplogroup context.

This finding contrasts with previous studies reporting on the absence of pathogenic mtDNA variants, especially including the m.3243A>G *MT‐TL1* variant, from Black African populations [10, 12, 25, 44, 59]. Importantly, most of these studies primarily focused on broad patient cohorts where PMD was not the only or even the most likely cause of disease. A more recent study that analyzed mtDNA sequences from whole exome sequencing data in 342 South African and 21 Zambian patients with undiagnosed neuromuscular disease (NMD) found no known causal pathogenic variants in any patients with L haplogroups. However, they did describe incidental findings in four patients with L0 haplogroups, including two patients with the m.1555A>G aminoglycoside induced deafness variant with no evidence of hearing loss and two patients with the m.14484T>C LHON variant with no associated LHON phenotype [12]. As their cohort included only undiagnosed NMD patients, it is plausible that the study may have excluded South African patients that had already received a diagnosis through local diagnostic services.

Although these data confirm that well‐described pathogenic mtDNA variants do occur in the context of disease in patients from African lineages, it is notable that only 57% of PMD mtDNA disease patients from the South African cohort and 9% of African American patients in the CHOP USA mtDNA‐based PMD clinical cohort had L haplogroups. These percentages are lower than expected considering that the 2022 South African census reported more than 81% of the population to be Black South Africans [60], and Black individuals with African American heritage are reported to account for at least 12.1% of the USA population [61]. These figures represent a significant underrepresentation of L‐haplogroups than expected in the South African cohort (57% compared with 81%, *p* < 0.0001). A similar discrepancy was reported in a North American cohort where less than 3% of diagnosed patients were reported to be African American [47], although it should be noted that many of the CHOP Mitochondrial Medicine patients analyzed here were also enrolled in that study. Such a pronounced disparity was not seen in the US data in our study (9% compared to 12.1%, *p* = 0.236), although numbers remain low.

Furthermore, given that the South African mtDNA genome data originates from the only diagnostic referral laboratory both in the country and in sub‐Saharan Africa, the overall diagnostic confirmation rate in both African and other lineages remains far below the 1 in 4300 minimal prevalence reported in different parts of the world [2, 62, 63, 64]. We postulate there are multifactorial reasons for this finding, including referral bias for mtDNA genome sequencing, lack of access to diagnostic testing, reduced clinical awareness of PMD in overburdened health systems with high prevalence of infectious diseases, and complex political and socio‐economic factors. While it is not known whether African haplogroup context influences the clinical disease penetrance of mtDNA pathogenic variants in carriers, the retrospective review of PMD cases presented here demonstrates that a wide range of African L haplogroups do manifest PMD from both common and rare mtDNA pathogenic variants.

The importance of haplogroup context in modifying phenotypic expression of PMD should not be ignored. A common population non‐pathogenic variant, m.13708G>A, normally associated with haplogroup J, was recently reported as a rare cause of PMD in a haplogroup H7A background [65]. Similarly, a study in African patients with Parkinson's disease suggested that haplogroup context is important in modifying the impact of population variants in the context of complex disease [66]. Interestingly, a study by Queen et al. reported that other mammals may carry known human pathogenic mtDNA variants in the absence of disease. They showed, for instance, that the m.3243A>G human pathogenic variant in *MT‐TL1* can be a frequent occurrence in other mammals without causing disease when in a single haplotype context with two other *MT‐TL1* variants (m.3253T>C and m.3254C>T) that are rare but do also occur in the human population. These variants were predicted to stabilize the mitochondrial tRNA^Leu^ molecule, which could modulate the effect of the m.3243A>G variant. The m.3253T>C variant has been associated with haplogroup L2b1a2, although it is not unique to this single sub‐haplogroup context [55, 58]. Despite low numbers, it is notable that none of the 11 m.3243A>G positive PMD families identified in this retrospective study carried this variant in the context of the African haplogroup L2b. Another study investigated whether confirmed human mtDNA pathogenic variants occur across 726 multiple sequence alignments derived from 33 non‐human vertebrate species. Fifty‐eight pathogenic human mtDNA variants were found to occur in the sequences of these species, with evidence of population variants in these animals that could mask the pathogenicity [49]. While some of these variants were present on the human phylogeny, others were not.

Our finding multiple high‐level and sub‐haplogroup contexts of causal mtDNA pathogenic variants in PMD patients proves that although a possible contributor, the modifying effects of haplogroup context are not a main driver of the diagnostic disparities evident for African patients. Rather, it is more likely that the majority of African mtDNA‐based PMD cases simply go undiagnosed for reasons other than genetic modifying factors. One such possible reason is the inaccessibility to and/or unavailability of specialized mtDNA genetic diagnostic services on the African continent. A recent study by Raga et al. showed that only a small percentage of African countries had access to local genetic testing services for NMDs in children, mostly limited to single gene testing for a subset of more common NMDs like spinal muscular atrophy and Duchenne muscular dystrophy. Although a number of countries in Africa had access to overseas referrals, more extensive testing is often not supported by public healthcare services in the majority of African countries [67]. This lack of access is further supported by the visitor and audience geolocation heat maps of the Mitomap.org [58] mtDNA variation database (https://clustrmaps.com/site/h7h5), suggesting that this globally recognized resource is not used to a great degree by African clinicians and medical geneticists.

Strikingly, only two of the 15 m.3243A>G positive cases in our combined cohorts presented with the milder MIDD phenotype typically associated with this variant. This suggests that only the most severe, multi‐system, syndromic cases come to the attention of specialized medical services in these settings. Socio‐economic and overburdened healthcare services may account for some of these discrepancies. Similarly, in the United States, there may be under recognition of PMD in patients of African origin, leading to lower rates of clinical referral to tertiary mitochondrial medicine centers and/or reduced frequency of mtDNA genome diagnostic testing.

The challenges faced by African researchers and healthcare workers in making sense of mtDNA variation as it relates to the health of Black South African populations were highlighted in a 2014 meeting report, resulting from a workshop aimed at addressing the diagnostic disparities in South Africa and globally [16]. Attendees included the core of the South African mitochondrial community, as well as contributors from the UK, with a focus on reviewing the current knowledge base while fostering a more structured collaborative approach to improving our understanding of PMD in Africa. The published meeting report and subsequent publications highlighted the limited existing capacity to provide equal and comprehensive diagnostic services to all affected patients as a key contributor to the diagnostic disparities seen in southern Africa [14, 16]. Healthcare programs in Africa are typically orientated towards the global challenges posed by preventable and infectious diseases. Access to specialist services, such as neuromuscular expertise, capacity for neuroimaging, biochemical investigation, and specialized genetic testing, is generally limited [67].

The findings from this study, when taken in the context of recent advances in our understanding of mtDNA variation in different haplogroup contexts, highlight the need for deeper investigations into the socio‐economic factors preventing diagnosis of PMD in African patients globally, as well as the presence of common haplogroup defining variants in unrelated haplogroups. As an example, a common European population variant might occur out of place in the L‐haplogroup context of undiagnosed individuals with suspected PMD; in this event, the European haplogroup marker should not be ruled out as having pathogenic potential. A good example of variants occurring in a novel haplogroup context and having a pathological effect is the recent description of a haplogroup J‐defining variant conferring a disease phenotype in the context of haplogroup H7A [65]. A prospective study of asymptomatic carriers for common mtDNA pathogenic variants would also be important to accurately inform the relative influence of L macro or microhaplogroups on clinical penetrance of PMD. We hypothesize that multiple undiscovered mtDNA‐related disease associations remain to be identified in African patients, which may broaden our understanding of this field. Indeed, much remains to be discovered in understanding the role of pathogenic variants in mitochondrial genes in human disease globally.

Overall, we have demonstrated that PMD due to known pathogenic mtDNA variants does occur in the context of multiple African (L) haplogroups and causes a similar spectrum of clinical phenotypes as have been reported worldwide. Yet, numbers of diagnosed black African ancestry cases remain low relative to expected population frequencies both in the South African state healthcare services and in the United States, although the latter did not reach statistical significance. We suggest that this may be due in part to diagnostic inequalities that still exist on both continents, but to a greater degree in Africa where resources are scarce. Haplogroup context may play a role in clinical disease penetrance and/or the clinical ability to recognize disease, and there may be pathogenic mtDNA variants yet to be discovered. Unraveling the role of haplogroup context and possible nDNA modifiers in phenotypic expression of disease in patients with Black African ancestry will assist in closing the diagnostic inequality gap in patients of African heritage globally. However, this should not distract from addressing diagnostic disparities caused by economic inequalities.

## Author Contributions

Conceptualization, planning, and design of this study was performed by S.M., G.T.R., M.J.F., and J.L.E. S.M., K.K., and S.D. performed SA cohort experiments. S.M. performed SA cohort analysis, data review, and manuscript preparation. G.T.R., D.B., J.L.E., and M.J.F. supervised the study. E.M.M., I.G.‐S., L.E.M., and M.J.F. performed USA cohort analysis and data review. All authors approved the final manuscript.

## Ethics Statement

Institutional ethical approval for this study was obtained from the UCT Human Research Ethics Committee (HREC/REF: 156/2021). Subjects from the United States were enrolled via an informed consent process or decedent waiver into Children's Hospital of Philadelphia (CHOP) Institutional Review Board (IRB) #6177 (Falk, PI).

## Consent

All procedures followed were in accordance with the ethical standards of the responsible committee on human experimentation (institutional and national) and with the Helsinki Declaration of 1975, as revised in 2000. Subjects from South Africa were enrolled via an informed consent process where possible, or through a consent waiver process approved by the UCT Human Research Ethics Committee (HREC/REF: 156/2021). Subjects from the United States were enrolled via an informed consent process or decedent waiver into Children's Hospital of Philadelphia (CHOP) Institutional Review Board (IRB) #6177 (Falk, PI).

## Conflicts of Interest

The authors declare no conflicts of interest.

## Data Availability

The data that support the findings of this study are not publicly available due to privacy or ethical restrictions.
